# The self-annealing phenomenon of electrodeposited nano-twin copper with high defect density

**DOI:** 10.3389/fchem.2022.1056596

**Published:** 2022-11-24

**Authors:** Haneul Han, Chaerin Lee, Youjung Kim, Jinhyun Lee, Sanghwa Yoon, Bongyoung Yoo

**Affiliations:** Department of Materials Science and Chemical Engineering, Hanyang University, Ansan, South Korea

**Keywords:** self-annealing, electrodeposition, nanotwins, low temperature, high current density

## Abstract

Electroplated copper was prepared under typical conditions and a high defect density to study the effect of the defects on its self-annealing phenomenon. Two conditions, grain growth and stress relaxation during self-annealing, were analyzed with electron backscattered diffraction and a high-resolution X-ray diffractometer. Abnormal grain growth was observed in both conditions; however, the grown crystal orientation differed. The direction and relative rate at which abnormal grain growth proceeds were specified through textured orientation, and the self-annealing mechanism was studied by observing the residual stress changes over time in the films using the sin^2^Ψ method.

## Introduction

As Moore’s law nears its physical limits, a new type of integrated circuit device with high density is needed for the Internet of things (IoT), artificial intelligence (AI), and other wearables (Knickerbocker et al., 2018). Therefore, to achieve smaller form factors, lower power, and high performance, advanced packaging technologies, such as a 3D interconnection, are promising solutions. In advanced 3D interconnection technologies, Cu-to-Cu direct bonding has received attention because of a lower interface resistance. Electroplated Cu has been mainly studied to connect the device electrically in the Cu-to-Cu bonding (McMahon et al., 2005; Beica et al., 2008; Gueguen et al., 2010; Radisic et al., 2011; Kong et al., 2012; Liu et al., 2015). (111)-oriented nano-twinned Cu for bonding has been widely investigated because of the fastest surface diffusivity and its properties (Juang et al., 2018; Chiu et al., 2021; Lu et al., 2021). In addition, nano-twinned Cu, which has a high density of nano-twin boundaries in its grain, has attracted attention with its ultrahigh strength, ductility, high electrical conductivity, and thermal stability (Lu et al., 2004; Shen et al., 2005; Chen and Lu, 2007; Saldana et al., 2011). However, the microstructure of electroplated Cu generally changes during room temperature storage, a phenomenon termed self-annealing. Therefore, preservation of the grain orientation and nano-twin structure prior to bonding are important for connecting the bonding interface at low process temperature.

Recrystallization and grain growth occur at room temperature during a self-annealing phenomenon, unlike the conventional annealing process at high temperatures between 600°C and 1000°C (Blaz et al., 1983; Huang et al., 1997; Seah et al., 1999; Hau-Riege and Thompson, 2000; Koo and Yoon, 2001; Militzer et al., 2004; Hasegawa et al., 2006; Stangl et al., 2008). The drastic change in electrical, mechanical, and crystallographic properties was accompanied by a self-annealing phenomenon (Ritzdorf et al., 1998; Brongersma et al., 1999; Ueno et al., 1999; Lagrange et al., 2000; Hara et al., 2003; Dong et al., 2008; Cheng et al., 2010; Huang et al., 2010).

Ho et al. conducted an *in situ* investigation of the self-annealing behavior of electroplated copper films with organic additives. Copper self-annealing was accelerated by increasing the current density, and grain growth was accompanied by an increase in the twin structure and a decrease in the grain orientation spread (Ho et al., 2016). In addition, Sung et al. proposed that the defect energy causes the recrystallization of the copper film, and it can be varied depending on the deposition type, which differs by the dynamic interaction of bis-(3-sulfopropyl) disulfide (SPS) and polyethylene glycol (PEG) during electrodeposition (Sung et al., 2017). Although many papers focused on the self-annealing behavior of Cu with electrodeposition and other methods, studies about the relationships between defects, especially nano-twin lamellae, and self-annealing behaviors have not been clearly represented.

In this paper, Cu with a high density of defects and nano-twin lamellae was electroplated based on our previous research. A lower electrochemical bath temperature and higher current density generated a high density of defects with nano-twin lamellae in the Cu (Lee et al., 2020; Han et al., 2021). Crystallographic defects are formed in every material to thermodynamically stabilize the system. The defects particularly affect the intrinsic properties of materials such as the mechanical properties (Lubomirsky, 2006; du Plessis et al., 2020; Liu et al., 2020). Especially for nano-twinned Cu, one of the defects has attracted great attention because of its mechanical and electrical properties (Lu et al., 2004; Shen et al., 2005; Chen and Lu, 2007; Saldana et al., 2011). This study compared the microstructure change over time with a typical self-annealing sample, room temperature Cu (RT Cu), and a nano-twinned Cu (NT-Cu) to investigate the effect of the defects on the self-annealing phenomenon. Specifically, the effect of defects in pure Cu electroplated without any additives on the self-annealing behavior was studied by monitoring the microstructural change over time. Electron backscattered diffraction (EBSD), a high-resolution X-ray diffractometer (HR-XRD), and residual stress analysis with X-ray diffraction (XRD) were used for various microstructural analyses. Residual stress analysis in the Cu film on the substrate was characterized by XRD according to the sin^2^Ψ method in which the theta angle was monitored with a variety of tilt angles (Bobet et al., 1995; Luo and Yang, 2017; Lin et al., 2020).

## Experimental

Electrodeposition of Cu was conducted in a jacketed beaker (inner diameter: 10 cm; outer diameter: 14 cm; height: 20 cm) filled with an electrolyte. The Cu layer was electrodeposited on a p-type heavily doped (100) Si substrate (<0.01 Ωcm) and 200 nm Cu/20 nm Ti seed layer, which were deposited with an electron beam (e-beam) evaporation system. The two-electrode experiment was conducted with a Pt-coated mesh (7 × 36 mm^2^) as a counter electrode and the Si wafer with a Ti and Cu seed layer covered with silicone masking tape to expose the specific area (10 × 10 mm^2^) as the working electrode.

Cu electrodeposition was performed with a pH 1.0 acidic electrolyte adjusted with 95% sulfuric acid (0.19 M), in which the solution comprised copper sulfate (1 M) without additives was in deionized water (resistivity: 18.6 MΩ). Two types of Cu films were prepared with a programmable DC power supply (Dawoo nanotech, DADP-20010R) and a thermostatic circulator (JEIO tech, RW-0525G) to control the electrolyte temperature.

Two types of Cu specimens were prepared with different electrolyte temperature, and the same solution and plating conditions. A high current density, 210 mA/cm^2^, was applied to the specimens to achieve self-annealing, which was accelerated by increasing the current density (Ho et al., 2016). However, the electrolyte temperature remained different, one was deposited at room temperature, 22.5°C, and the other at 0°C. The rotating speed was maintained at 500 rpm with an impeller coated with a stop-off lacquer to prevent other unnecessary reactions.

Crystallographic evolution during self-annealing was analyzed with EBSD operated at 20 kV, which was performed with field emission gun scanning electron microscope (FEG-SEM, 7100F, JEOL, Japan) combined with a TEAM™ PEGASUS EBSD system (EDAX, USA). The grain and grain boundary analysis were characterized with OIM™ software (EDAX). An electropolishing process was conducted for EBSD and residual stress analysis to flatten the surface of the electroplated Cu with 85% phosphoric acid. Electropolishing of Cu was applied at 4 C with a potentiostat/galvanostat (Versa STAT 4, Princeton Applied Research, USA) at room temperature.

HR-XRD (SmartLab, Rigaku, Japan) was used to characterize the preferred grain orientation of the Cu and operated at 40 kV and 30 mA from 35° to 75°. To analyze the residual stress, XRD (PANalytical; Expert pro-MPD, Almelo, Netherlands) was operated with a Cu-Kα (*λ* = 0.15418 nm) source generated at 40 kV and 40 mA (spot size = 3 × 3 mm^2^). A sin^2^Ψ method was analyzed in the (111) grain orientation over time with X’Pert Stress software (Baczmanski et al., 2002; Delbergue et al., 2016; Luo and Yang, 2017).

A cross-sectional image was observed by a focused ion beam (FIB, LYRA 3 XMH, TESCAN, Czech Republic). A cross-sectional sample for FIB analysis was prepared with the FIB milling performed with a small ion beam dose (current = 10 nA, voltage = 30 kV), and ion channeling contrast (current = 30 pA, voltage = 30 kV) was used to image the grains on the cross-sectional plane.

## Results and discussion

**FIGURE 1:**
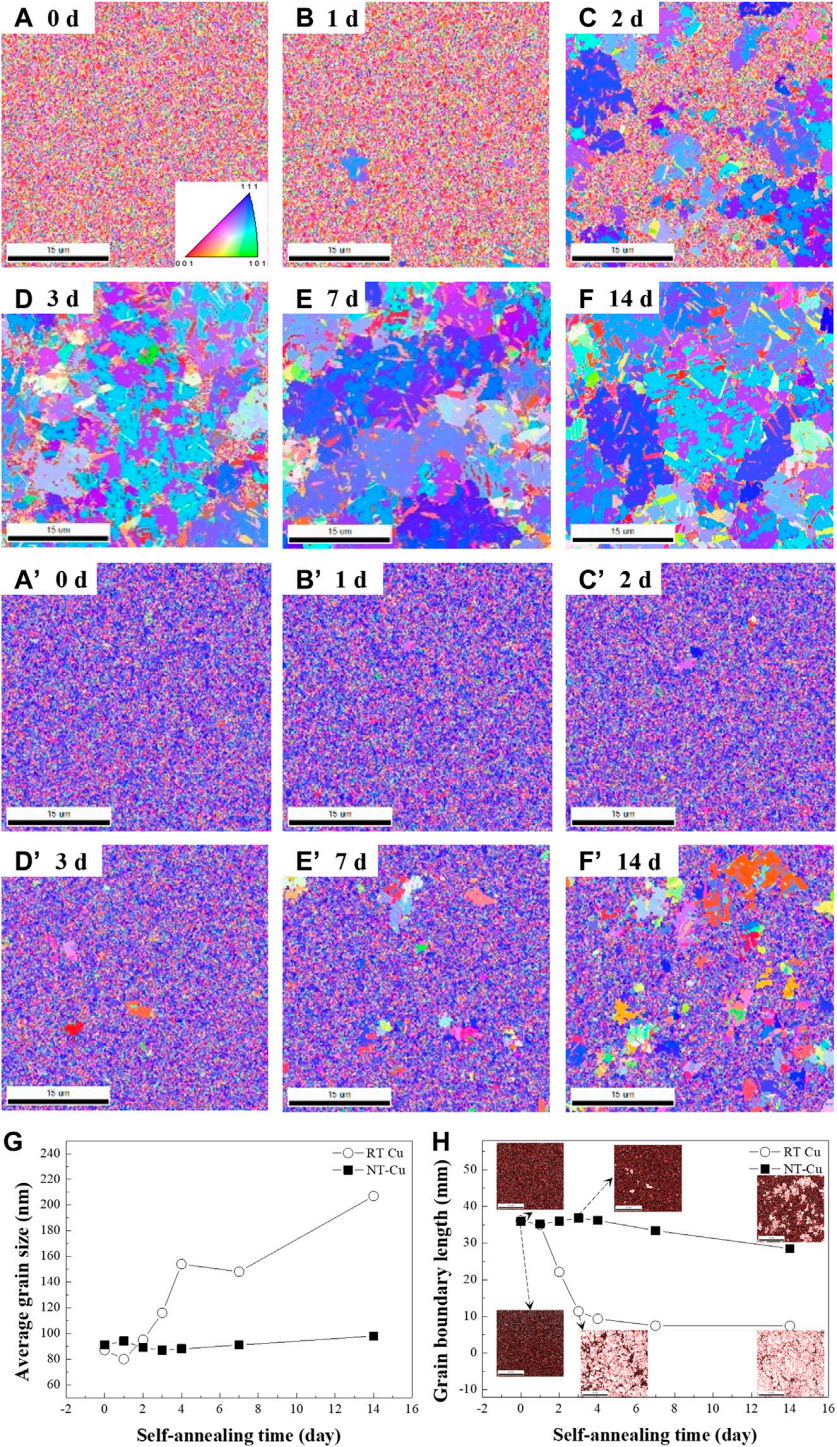
**(A–F)** EBSD inversed pole figures (IPFs) of the RT Cu plating at **(A)** 0, **(B)** 1, **(C)** 2, **(D)** 3, **(E)** 7, and **(F)** 14 days. **(A’–F’)** IPFs of NT-Cu at the same time as **(A–F)**. **(G)** Average grain size and **(H)** grain boundary length evolution with time for RT Cu and NT-Cu.

Crystallographic analysis was performed over time using the EBSD and HR-XRD measurements. To verify the surface crystallographic evolution, the surface grain orientation and morphologies were characterized with EBSD analysis. The variation of the surface grains with respect to the electrolyte temperature under a current density of 210 mA/cm^2^ is represented in Figures 1A–F’. Grain growth of the sample electroplated at 22.5°C (RT Cu) occurred, and the overall grain orientations changed within a week, as shown in Figures 1A–F. Alternatively, Cu electroplated at 0°C (NT-Cu) experienced abnormal grain growth over time, and the growth was not saturated, as shown in Figures 1A’–F’. Figures 1A–F shows that the RT Cu grains with random orientation grew to the (111) orientation over time, and Figures 1A’–F’ shows the change of the NT-Cu grains that grew from the (111) preferred orientation to other orientations. As shown in Figures 1A–F’, abnormal grain growth occurred in both specimens regardless of the condition.

**FIGURE 2:**
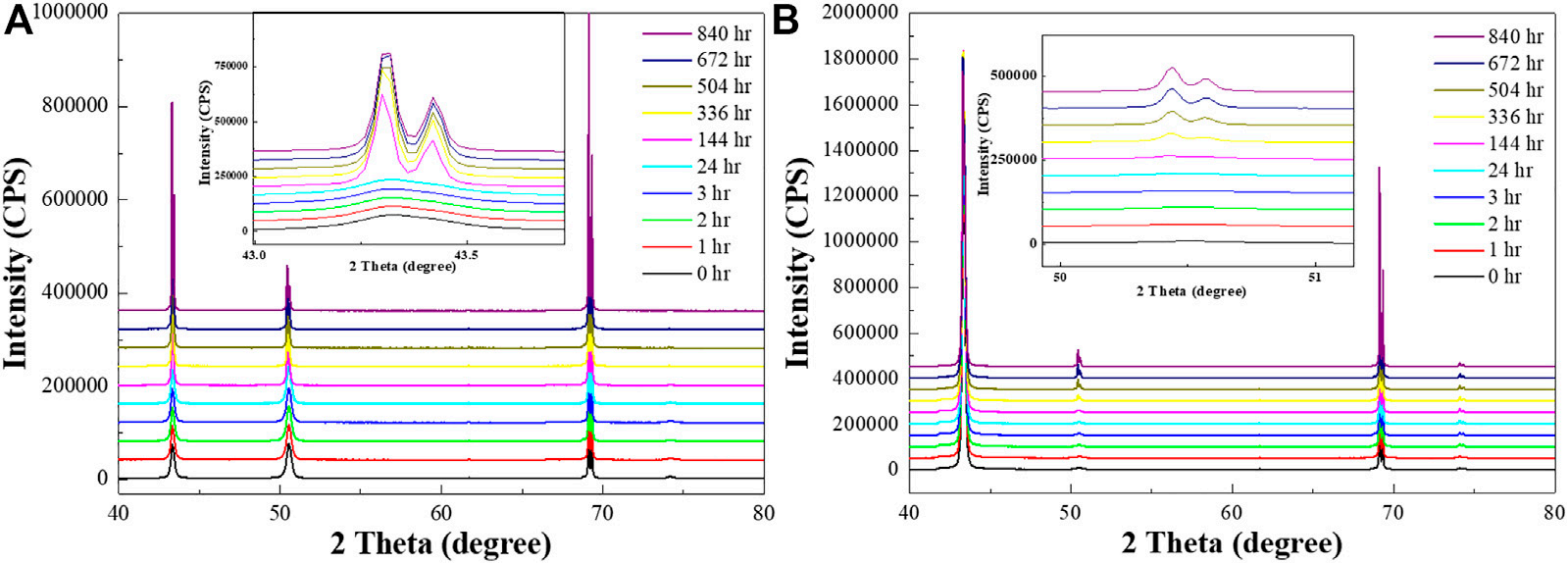
HR-XRD profiles of the **(A)** RT Cu and **(B)** NT-Cu films at *t* = 0 (black), 1 h (red), 2 h (green), 3 h (blue), 24 h (cyan), 144 h (magenta), 336 h (yellow), 504 h (dark yellow), 672 h (navy), and 840 h (purple).

HR-XRD analysis was used to study the grain orientation and texture change tendency with both conditions of Cu by time (Figure 2). Three major peaks in Cu were measured in Figure 2, corresponding with the Cu peak in PDF #03-065-9026. Figure 2A shows an increase in the (111) grain orientation peak in RT Cu, especially after one week. However, there is no drastic change in the peaks over time in NT-Cu (Figure 2B); the (200) peak appeared gradually.

**FIGURE 3:**
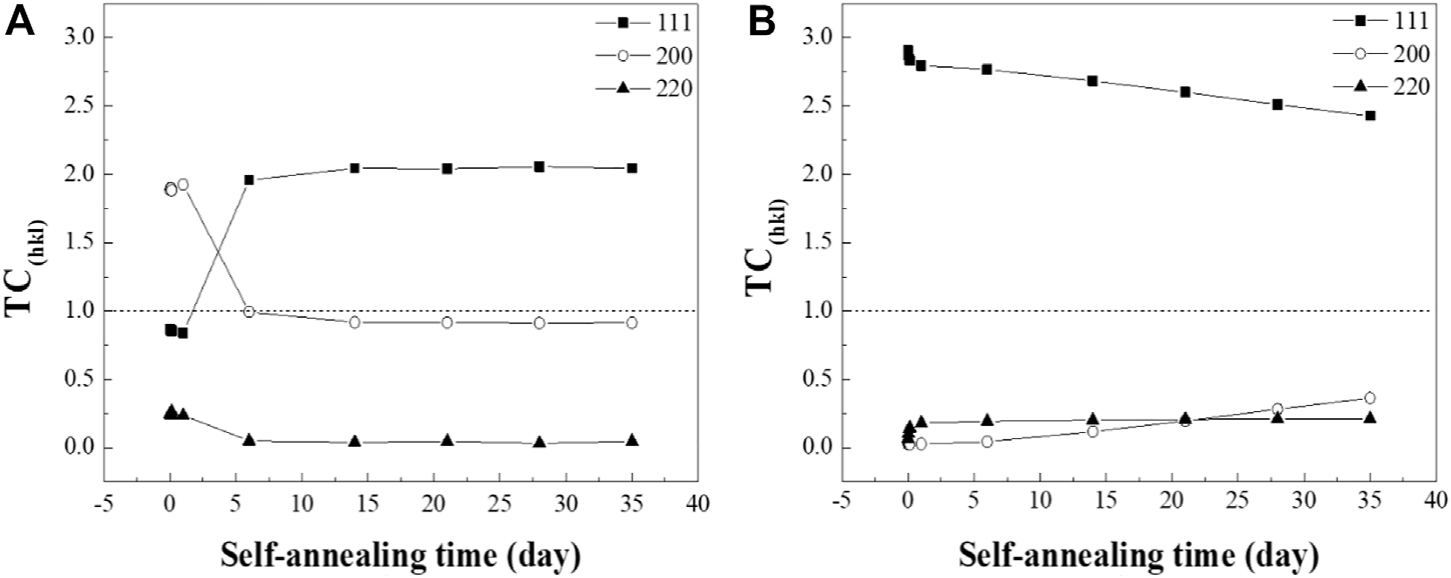
TC_(hkl)_ evolution of the **(A)** RT Cu and **(B)** NT-Cu films calculated with the (111), (200), and (220) orientations.

To elucidate the texture changes of the specimens, the texture coefficient (TC) was calculated by Eq. 1, which was measured by HR-XRD analysis (Dixit et al., 2007; Hamid and Aal, 2009).

$$TC\left(hkl\right)=\frac{I\left(hkl\right)}{I_{0}\left(hkl\right)}/\frac{1}{n}\sum \frac{I\left(hkl\right)}{I_{0}\left(hkl\right)}$$ (1)

where I(hkl) is the intensity of the samples in the experiment, I_o_(hkl) is the standard relative intensity of the peak from PDF #03-065-9026, and n is the total number of peaks analyzed. Three prominent peaks of Cu, the (111), (200), and (220) orientations were applied to calculate the TC values. TC > 1 refers to the preferred orientation of the materials (Figure 3).

The (200) orientation of the RT Cu was dominant in the initial stage of the self-annealing, while RT Cu had the random orientation depicted in Figure 3A. Interestingly, a drastic transition from the (200) to (111) orientation was observed in the RT Cu within a week. Alternatively, the crystallographic evolution of NT-Cu was observed slowly over a long period (Figure 3B). The (111) oriented grains were illustrated as the preferred orientation of the NT-Cu during self-annealing. However, the (111) peak decreased over time while the (200) peak increased. In other words, abnormal grain growth of NT-Cu progressed with the grains in the (200) planes, while that of the RT Cu progressed with the grains in the (111) planes.

As shown in Figure 1G, both as-deposited RT Cu and NT-Cu had nanocrystalline grains with an average grain size of 87 nm and 91 nm. A high free energy per unit volume of the grain was formed because of the increase in the grain boundary area (Park et al., 2017). Grains of both RT Cu and NT-Cu were driven to grow at room temperature to reduce their internal energy because of their high grain boundary energy (Yang et al., 2019).

In the case of RT Cu, grains relatively superior to the (200) orientation in the initial stage of self-annealing changed into the (111) orientation to release the initial high internal energy. Grains with (111) planes parallel to the plane of the film have the lowest surface energy in the face-centered cubic (FCC) structure, which results in grain growth in a direction (Foiles et al., 1986; Udler and Seidman, 1996; Jian-Min et al., 2004; Wen and Zhang, 2007; Fishman et al., 2013). Additionally, (111) planes are conventionally known as the plane with the fastest growth rate (Thompson, 1990; Lee et al., 2001), which causes grains in RT Cu to grow rapidly in the (111) orientation. However, grains in NT-Cu grew into the (200) orientation, with the minimum strain energy in the FCC metals (Zielinski et al., 1995; Lee, 2000; Zhang et al., 2002; Zhang et al., 2005). Although the initial average grain size generated a similar grain boundary energy in both Cu, a strain energy relaxation mechanism occurred in NT-Cu. As confirmed in the previous research, NT-Cu has a high density of defects on its surface even though it has a high density of nano-twin lamellae in its vertical grains. The defects increase the internal strain energy, causing grain growth in the direction of less strain energy (Lee et al., 2020; Han et al., 2021).

**FIGURE 4:**
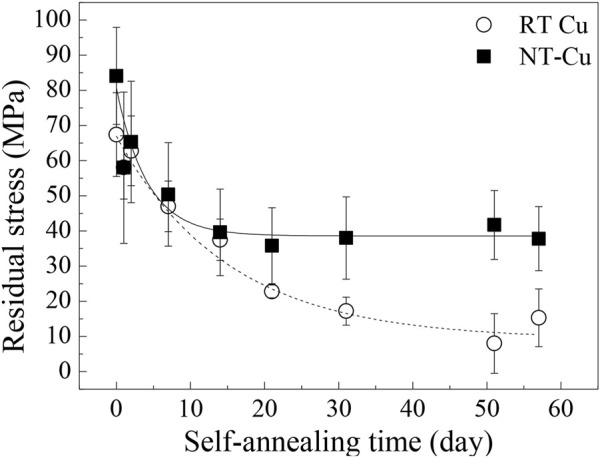
Residual stress evolution of the RT Cu and NT-Cu films over self-annealing time.

The sin^2^Ψ method by X-ray diffraction (XRD) was used to verify the residual stress of RT Cu and NT-Cu in the unidirectional direction over time, as shown in Figure 4. By detecting the theta value (*?*) according to the psi value (Ψ), residual stress in the sample was calculated by elastic modulus and Poisson’s ratio of bulk Cu (Baczmanski et al., 2002; Delbergue et al., 2016; Luo and Yang, 2017).

Figure 4 shows the residual stress changes in RT Cu and NT-Cu during self-annealing. The stress in the RT Cu was calculated as 67.4 MPa in the initial stage of self-annealing and gradually decreased to approximately 8.0 MPa after 51 days. In its initial state, NT-Cu has a relatively higher stress than RT Cu (84.1 MPa), and it rapidly reduced and saturated to approximately 40 MPa, approximately 47% of the initial stress. Stress changes during self-annealing have been conventionally expressed as a stress evolution of Cu, divided into two categories: stress generation because of grain growth and stress relaxation by dislocation plasticity (Brongersma et al., 1999; Harper et al., 1999; Huang et al., 2010). Figure 4 shows that high residual stress existed in both conditions as compared to other references where it existed at the initial stage of the self-annealing (Brongersma et al., 1999; Lagrange et al., 2000).

Both conditions had a similar average grain size and grain boundary length in the initial state shown in Figures 1G,H; however, the initial stress of the two conditions differed by approximately 16.7 MPa. Internal strain generated by defects can cause stress in the metal (Dieter and Bacon, 1976). The initial internal strain caused by the grain boundary was similar, so it is expected that the initial defect density difference between the two conditions generated the residual stress difference (Basu et al., 2017). Stress saturation in the NT-Cu can be supposed that the stress reached the self-diffusion equilibrium because of its defects, while RT Cu lost most of its stress and only remained approximately 10 MPa after self-annealing (Figure 4). The self-diffusion of RT Cu occurred actively over time.

Chen et al. investigated the effect of triple junctions generated with a grain boundary and twin boundary on atomic diffusion (Chen et al., 2008). The twin boundary separates the grain into two sub-grains and generates the triple junction with the grain boundary. In their research, the atomic diffusion was delayed in the triple junction between the grain boundary and twin boundary.

**FIGURE 5:**
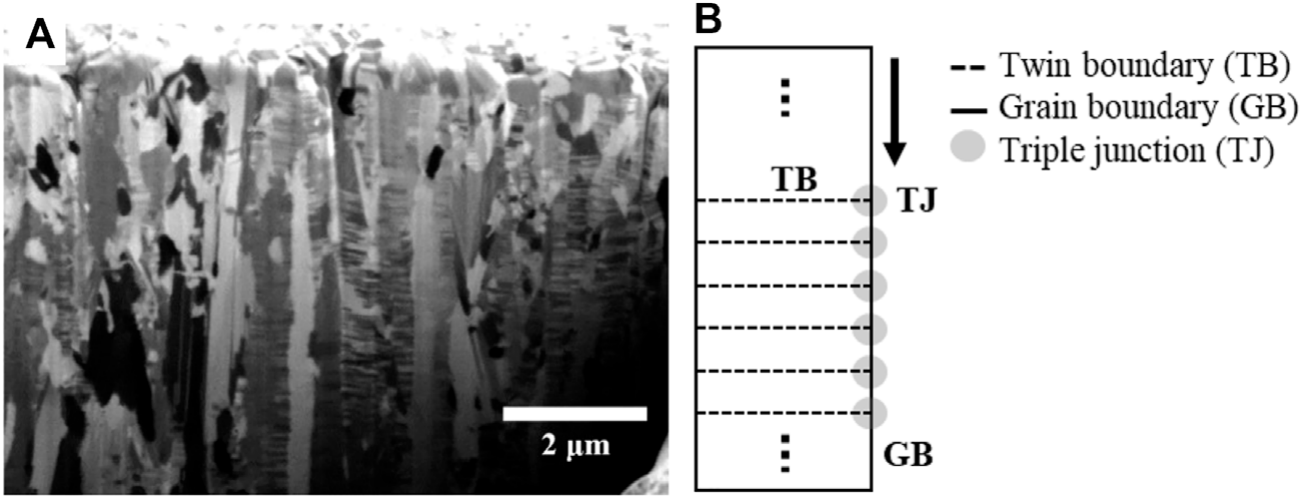
**(A)** Cross-sectional FIB image of as-deposited NT-Cu and **(B)** schematics of the nano-twin column with nano-twin lamellae, grain boundary, and triple junctions.

Figure 5A shows the high density of the nano-twin lamellae in columnar grains of the as-deposited NT-Cu. In this study, NT-Cu has a high density of nano-twin lamellae, resulting in many triple junctions between the grain boundary and twin boundary, similar to Figure 5B. The grain growth phenomenon occurs *via* atomic diffusion. Atoms mainly diffuse along the intergranular path, called grain boundary diffusion (Nieh and Nix, 1980; Kolobov et al., 2001). Therefore, self-diffusion *via* the grain boundary can be illustrated as shown in Figure 5B. It was expected that atoms diffused along the grain boundary and met the innumerable triple junctions that disturbed the diffusion. In other words, Cu atoms were diffused at both conditions; however, numerous triple junctions in the NT-Cu delayed their motions. The grain growth rate of NT-Cu is slow because of the triple junction delay.

## Conclusion

The effect of the high defect density and nano-twin lamellae on the self-annealing phenomenon was investigated by analysis of the grain morphology, orientation, texture, and residual stress. Grains of NT-Cu grew to the (200) orientation, while RT Cu grew to the (111) orientation according to EBSD and HR-XRD analysis. Both conditions had a similar average grain size; however, their crystalline evolution patterns and rates differed. In particular, RT Cu grains grew and saturated within a week, whereas NT-Cu grains grew gradually and did not saturate after a month. However, the opposite tendency was observed in residual stress. Residual stress in RT Cu gradually decreased over time, and the stress in NT-Cu was dramatically reduced in the initial stage of self-annealing and saturated at 50%. As a result, the high defect density contributed to the grain growth texture and the nano-twin lamellae contributed to the self-diffusion rate at room temperature.

## Data Availability

The original contributions presented in the study are included in the article/Supplementary material, further inquiries can be directed to the corresponding authors.
